# Pricing a Protest: Forecasting the Dynamics of Civil Unrest Activity in Social Media

**DOI:** 10.1371/journal.pone.0139911

**Published:** 2015-10-06

**Authors:** Brian J. Goode, Siddharth Krishnan, Michael Roan, Naren Ramakrishnan

**Affiliations:** 1 Discovery Analytics Center, Virginia Tech, Arlington, VA, United States of America; 2 Dept. of Mechanical Engineering, Virginia Tech, Blacksburg, VA, United States of America; 3 Dept. of Computer Science, Virginia Tech, Blacksburg, VA, United States of America; University of Warwick, UNITED KINGDOM

## Abstract

Online social media activity can often be a precursor to disruptive events such as protests, strikes, and “occupy” movements. We have observed that such civil unrest can galvanize supporters through social networks and help recruit activists to their cause. Understanding the dynamics of social network cascades and extrapolating their future growth will enable an analyst to detect or forecast major societal events. Existing work has primarily used structural and temporal properties of cascades to predict their future behavior. But factors like societal pressure, alignment of individual interests with broader causes, and perception of expected benefits also affect protest participation in social media. Here we develop an analysis framework using a differential game theoretic approach to characterize the *cost* of participating in a cascade, and demonstrate how we can combine such cost features with classical properties to forecast the future behavior of cascades. Using data from Twitter, we illustrate the effectiveness of our models on the “Brazilian Spring” and Venezuelan protests that occurred in June 2013 and November 2013, respectively. We demonstrate how our framework captures both qualitative and quantitative aspects of how these uprisings manifest through the lens of tweet volume on Twitter social media.

## 1 Introduction

Online social networks, such as Twitter, are open platforms for rapidly transmitting information about events observed by user populations. These networks are a rich source of data with potential explanatory and forecasting power. Examples of such studies using social media include financial interests [1, 2], disease spreading [3, 4], and protest activity (civil unrest) [5–7]. Our study builds on protest event-based diffusion models having foundations laid by Oliver and Myers [8]. There are a number of recent large-scale protests with events leaving footprints in social media. The Arab Spring, Brazilian Spring, and Venezuela online protest movements have been used as a canvas by many researchers to model civil unrest evolving in social media [9–11], along with studies of other high impact movements [12, 13]. Our focus is information propagation cascades [14] on Twitter for protest events occurring in Brazil and Venezuela. The Brazilian protests [15] (e.g. “Brazilian Spring”) in June, 2013 originated in the city of Saõ Paolo. These protests started by disputing the increase in public transportation fares, but quickly expanded to address other facets of governmental corruption. The Venezuelan protests were in opposition to their President in November, 2013 (e.g. see [16] for events leading up to his rule by decree). These were a precursor to the “Venezuelan Spring” protests that encompassed broader economic issues between February and June, 2014.

The dynamics of cascade growth is a complex problem characterized by many variables on multiple scales. A simple model of information diffusion is the Susceptible-Infected-Recovered (SIR) model [17] where each interaction is independent. Goel et al. [18] show deviations from the SIR structure in real networks containing many small cascades and few large cascades. Thresholding models such as [19, 20] have been developed to address the strength of network connections as a parameter for transmission. Analyses of the underlying network structure [21, 22] and temporal complexity [23] have resulted in numerical and analytic growth models of diffusion. However, forecasting cascade growth on social media using machine learning techniques adds further challenges. Many studies employ a traditional approach such as regression [24, 25] or classification [26, 27]. There are also a number of forecasting measures to consider such as popularity [28] in Digg, re-tweeting on the Twitter network [29], and user interests in microblogs [30, 31]. We use the recent work by Cheng et al. [32] of predicting photo reshare cascade sizes on Facebook as a template for our analysis.

The Twitter dataset we analyze consists of over 40 million tweets and 2 million users in South America. We filtered the tweets using a multi-lingual list of 961 protest related keywords such as “protesta”, “vigilantes”, and “vandalismo” and formed the cascades using a follower-network. Conventional network analysis (see [33]) has primarily considered structural and temporal (dynamic) properties of cascades. Structural features such as degree distribution and connectivity do tend to perform well on our data, but are not trivial to compute. The Twitter follower-graph used in this work has a node order of millions and edge order of billions. The dynamic nature of the cascades presents significant challenges in recomputing the structural features of this magnitude. A single computation of the Brazil Twitter graph’s structural features requires around half a terabyte of memory, and takes on the order of days to accomplish. In contrast, temporal features on the same data set can be stored and computed on an ordinary machine with 4 gigabytes on the order of hours. We improve on vanilla temporal features by representing Twitter cascade behavior in terms of a finite number of parameter sets. This parameter model draws heavily from game theory [34], especially the differential games framework proposed by Rufus Isaacs and Antony Merz [35, 36] that accounts for temporal evolution in decisions or choices [37]. A differential game models rational maximizing and minimizing agents optimizing over the same cost (payoff) function at each point in time. Although rationality is not guaranteed in a population, modeling it as such serves as a good measure of the degree to which behavior is rational with respect to a specific cost. Reluga [38] uses a differential game theoretic approach to study social distancing during an epidemic by modeling the cost associated with such distancing. We adopt a similar approach wherein we model a user’s interaction with a protest information cascade using a similarly conceived *cost of participation*. The assumption is that individuals will experience a cost associated with joining a Twitter cascade, and that this cost can be used to classify the behavior of the cascade as it evolves in time.

Our work adds a new wrinkle to such prediction problems by studying the notion of *cost of participation* with a differential game model. However, this work is not blind to the challenges that face forecasting cascade growth. There are multiple ways to evaluate a forecasting algorithm. Our method seeks to identify groupings of behavior, which perform very well given the cost model. Applying this to a more event specific metric, such as the number of tweets without the grouping, will likely result in decreased fidelity. However, the advantage of this approach is two-fold, because it is not resource consuming to extract the features, and the paradigm is general enough in its approach to represent cascades as data-driven behavioral classes. Additionally, the complex and interwoven consequences of actions can result in rather counter-intuitive results (i.e. [39]). Twitter is an expedient and uncertain environment with respect to human actions. Forecasting methods become plagued with high sensitivity to antecedent features, if known, subject to high levels of noise. Consequent forecasting where no prior information is known becomes intractable, time varying, and sensitive to external factors not captured in our temporal feature set. The scope of this work focuses on the more tractable approach of using a temporal differential game model to improve the classification of a cascade given a library of behaviors.

## 2 Analysis and Methods

### The Model

The proposed framework models activity cascades [14], one of many possible ways to define cascades in Twitter. When an individual posts a relevant tweet at time *t* and within a short duration, Δ, a few of their followers post a relevant tweet, we add these tweets to the cascade. A relevant tweet is one containing keywords from the keyword list given in the supplementary data. This process repeats until the cascade cannot be expanded any further. Note that the follower network for the users must be known to complete this process. We use the susceptible-infected-recovered (SIR) model to capture this intuition of cascade growth wherein individuals of a fixed population (of tweets or users) are believed to be in one of three states:

- *Susceptible*, *x*_*s*_: individuals that have not joined the cascade, but could potentially do so
- *Infected*, *x*_*i*_: individuals that have joined the cascade and are capable of influencing the susceptible individuals
- *Recovered*, *x*_*r*_: individuals that have left the cascade (e.g. now participate in another cascade)

**Fig 1:**
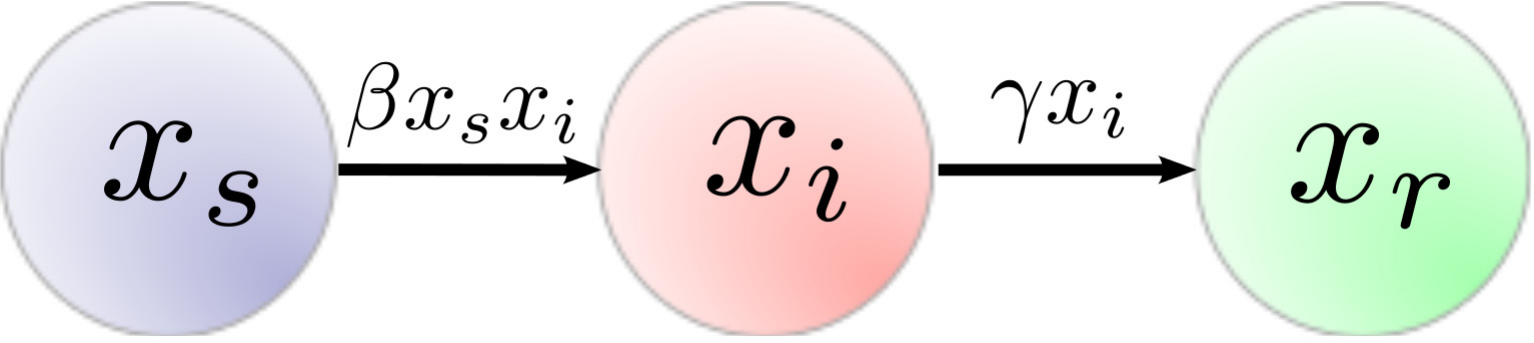
The state diagram for the SIR model. The nodes, *x*_*s*_, *x*_*i*_, and *x*_*r*_ represent counts within a population to the susceptible, infected, and recovered states, respectively. The transmission rate, *β*, controls the flow of susceptible individuals to infected. The recovery rate, *γ*, controls the flow of individuals out of the cascade.

Shown in Fig 1 are two parameters governing the rate at which individuals join one of the three states. The transmission rate, *β*, is the ratio of susceptible individuals that join the cascade by contact with members of the infected state. The recovery rate, *γ*, is the ratio of infected individuals that leave the cascade. The complete SIR model dynamics are a set of coupled nonlinear differential equations,

$$\begin{array}{c} {\dot{x}}_{s}=-\beta x_{s}x_{i} \end{array}$$ (1)

$$\begin{array}{c} {\dot{x}}_{i}=\beta x_{s}x_{i}-\gamma x_{i} \end{array}$$ (2)

$$\begin{array}{c} {\dot{x}}_{r}=\gamma x_{i} \end{array}$$ (3)

with initial conditions subject to the constraint, *x*_*s*_(*t*_0_) + *x*_*i*_(*t*_0_) + *x*_*r*_(*t*_0_) = *N*. The parameter, *N*, is the population count of individuals, and the constraint holds for all *t*. The cumulative cascade population, *s*, that has joined the cascade by time *t* is given by the individuals that have left the susceptible state, and are either currently infected or recovered.

$$\begin{array}{c} s\left(t\right)=N-x_{s}\left(t\right) \end{array}$$ (4)

Cost considerations wherein the dynamics at the population level can deviate from SIR dynamics Eq (3) are formed in the general framework where the states, $x\in X\subset {\mathbb{R}}^{3}$, evolve as

$$\begin{array}{c} \dot{x}=f\left(x,d\right)=f_{SIR}\left(x\right)+f_{D}\left(x,d\right) \end{array}$$ (5)

where *x* = [*x*_*s*_, *x*_*i*_, *x*_*r*_]^*T*^. We assume that the SIR dynamics, *f*_*SIR*_, are subject to a population level disturbance, *f*_*D*_(*x*, *d*). The separability of the disturbance term implies that the SIR model is always present to some degree. Examples of disturbances related to protests include portions of the population distancing themselves from the cascade by reducing their social networking time or bowing to societal pressure not to comment on a particular event. The SIR model of protest dynamics, Eq (3), is inserted into Eq (5), and the disturbance is given a yet unknown function *σ*

$$\begin{array}{c} \left[\begin{array}{c} {\dot{x}}_{s}\\ {\dot{x}}_{i}\\ {\dot{x}}_{r} \end{array}\right]=\underset{f_{SIR}}{\underset{︸}{\left[\begin{array}{c} \beta x_{s}x_{i}\\ \beta x_{s}x_{i}-\gamma x_{i}\\ \gamma x_{i} \end{array}\right]}}+\underset{f_{D}}{\underset{︸}{\sigma (x,d)}} \end{array}$$ (6)

where *d* is the population control input for *f*_*D*_ = *σ*(*x*, *d*). The only requirement placed on the disturbance function is *σ*(*x*, *d*) ∈ 𝒞^1^. The infinite horizon cost functional by which a population chooses *d* has the form,

$$\begin{array}{c} J\left(x,t,d\right)=\int_{t}^{\infty }g_{\sigma }\left(x,d\right)dt \end{array}$$ (7)

where *g*_*σ*_ is the running cost. Eq (7) is the accrued penalty of disturbing the cascade with *d* subject to the *σ* function at a given state. The population minimizes Eq (7).

**Fig 2:**
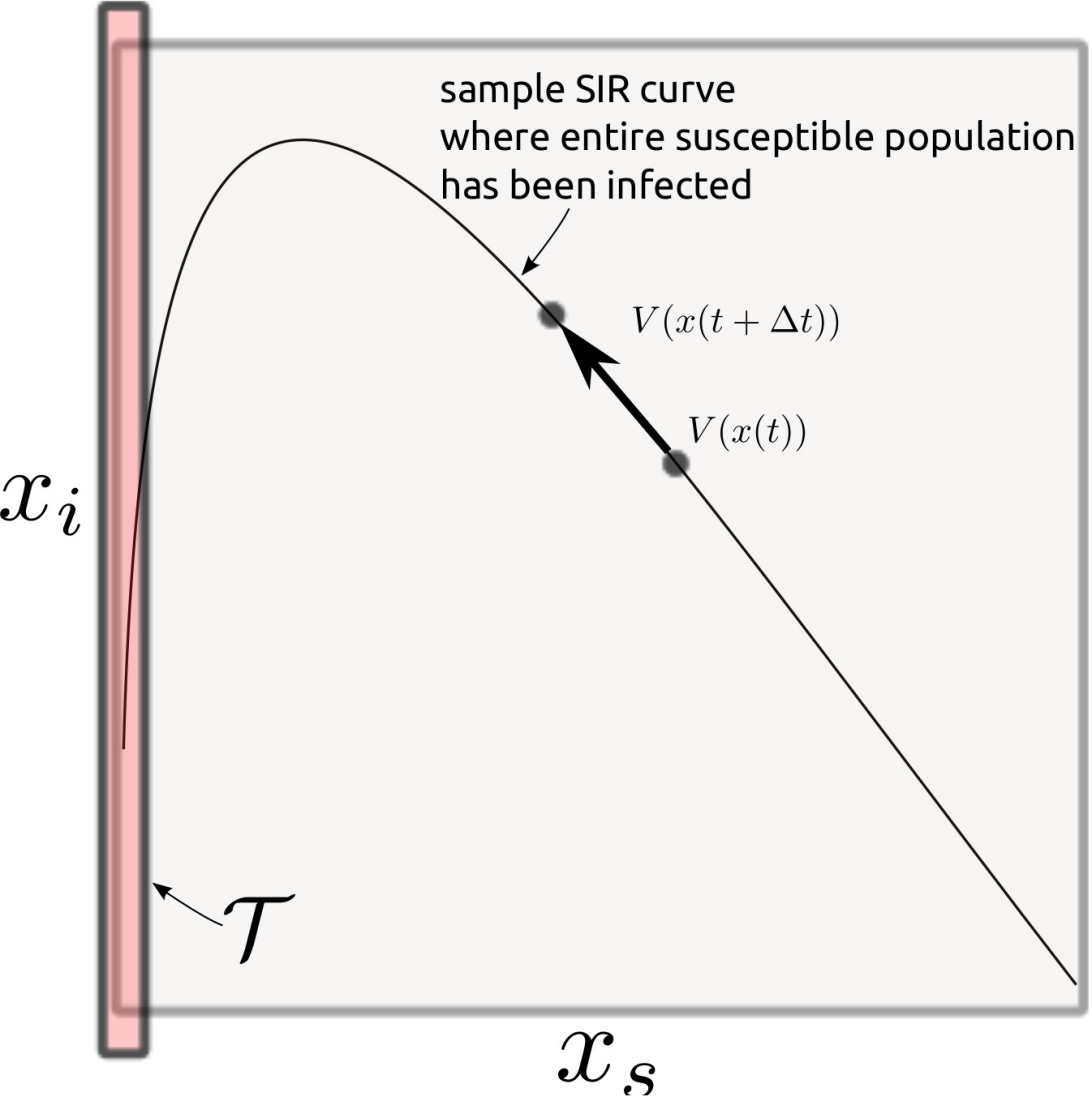
The *x*_*s*_, *x*_*i*_ phase plane showing a trajectory. The box on the left of the diagram is the target set, 𝒯, to which the state is directed. The function, *V*(*x*), measures the cost to go from any *x* to 𝒯. *V*(*x*) is decreasing along trajectories as *t* increases. The disturbance control, *d*, needed to guide the trajectory to 𝒯 can be found using these functions.

As shown in Fig 2, the solution to this equation consists of finding an optimal disturbance control, d*, and a corresponding value function, *V*(*x*(*t*)), such that *V*(*x*(*t*)) = *J*(*x*, *t*, d*). The value function represents the “cost-to-go” for reaching the target set, 𝒯 using control, d*. The target set and boundary conditions are identified as,

$$\begin{array}{c} \forall x\in 𝒯\subset X,V\left(x\right)=c_{0} \end{array}$$ (8)

where 𝒯 is a set of target states that are assigned the terminal cost, *c*_0_. In the case of information spreading in a social network, the target set for a population trying to reach the most number of users is 𝒯 = {*x* ∈ *X*∣*x*_*s*_ = 0}, shown in Fig 2. From Eq (4), this terminal condition assigns the target as reaching the entire *N* count population. Implicit in the preceding analysis are assumptions of (i) no finite end time of the cascade or outside intervention on the population level (i.e., individual participation only) and (ii) agents operating rationally with full knowledge per the cost structure. The first assumption is addressed by setting *c*_0_, so the state remains in the target set upon entry. The second assumption differentiates between cascades displaying different cost features given below.

We solve for *d* using the Hamilton-Jacobi-Bellman (HJB) equation [40],

$$\begin{array}{c} 0=\min_{d\in D}[g_{\sigma }\left(x,d\right)+\nabla_{x}V\left(x\right)f\left(x,d\right)]+\dot{V}\left(x\right) \end{array}$$ (9)

This is a continuous version of the equation, and the control, *d*, is found by,

$$\begin{array}{c} d^{*}=\underset{d\in D}{arg min}[\nabla_{x}V\left(x\right)f\left(x,d\right)] \end{array}$$ (10)

To solve numerically using the Fast Marching Semi-Lagrangian (FMSL) method [41], a discretized version of the HJB equation is given as,

$$\begin{array}{c} V\left(x_{j}\right)=\min_{d_{k}\in \tilde{D}}\{V\left(x_{j}+hf\left(x_{j},d_{k}\right)\right)+hg_{\sigma }\left(x_{j},d_{k}\right)\} \end{array}$$ (11)

where $x_{j}\in \tilde{X}$ is a discretization over *X*, *h* is the discrete fixed time interval, and $d_{k}\in \tilde{D}$ is the discretized control set. The FMSL solution yields the value function, *V*, and an associated control sequence

$$\begin{array}{c} d^{*}\left(x_{j}\right)=\underset{d_{k}\in \tilde{D}}{arg min}[\nabla_{x}V\left(x_{j}\right)f\left(x_{j},d_{k}\right)] \end{array}$$ (12)

There is an optimal control, d*, at every state in the grid lattice, and it depends on *g*_*σ*_.

We now incorporate the propensity of individual actions into the SIR framework using a Markov probability model. An individual is a member of the population, *i* ∈ {1, 2, 3, …, *N*}. The dynamics applied to the individual are uncertain transitions of *i* being a member of the susceptible, infected, or recovered populations at a given time. Using the proposed formulation in [38], the individual stochastic model is

$$\begin{array}{c} \dot{p}=Q\left(t;\hat{d}\right)p \end{array}$$ (13)

where $\hat{d}$ is the individual disturbance term and *p* = [*p*_*s*_, *p*_*i*_, *p*_*r*_]^*T*^ are probabilities of the individual belonging to a particular state. The transition-rate matrix is given by

$$\begin{array}{c} Q\left(t;\hat{d}\right)=[\begin{array}{ccc} -\pi \left(x,\hat{d}\right)\beta x_{i} & 0 & 0\\ \pi \left(x,\hat{d}\right)\beta x_{i} & -\gamma & 0\\ 0 & \gamma & 0 \end{array}] \end{array}$$ (14)

where the SIR parameters, *β* and *γ* follow from Eq (3). The function, $\pi (x,\hat{d})$, is a multiplicative adjustment to the transmission ratio. Combining the population and individual dynamics, the Bellman equation for finding the Nash equilibrium of an individual control $\hat{d}$ with population control *d* is shown [42] to be

$$\begin{array}{c} -\dot{V}=\left(Q^{T}-hI\right)V+g_{\sigma } \end{array}$$ (15)

where *V* is the individual value function, *h* is the discount factor, and *g*_*σ*_ is the running cost defined initially in Eq (7). The individual optimal strategy for cascade participation, $\hat{d}$, minimizes the individual contribution to the cost. In this framework, the individual observes the population dynamics in Eq (6), chooses $\hat{d}$, which subsequently adjusts the population level disturbance, *d*, until a Nash equilibrium is reached. No individual can do better by deviating from the population strategy, $d=\hat{d}$.

To solve the game, we show that an individual using $\hat{d}=d^{*}$ with no cost discounting will not deviate from the population optimal control, d*, in Eq (12). Furthermore, we can simplify the solution of the game when the population control d* is guaranteed to be a Nash equilibrium if ∃*d* *s*.*t*. ∀*x* ∈ *X*, ∣*σ*(*x*, *d*)∣ > ∣*βx*_*i*_ *x*_*s*_∣. To show this, we only need to find when Eqs (15) and (9) are equivalent under these conditions. This can be done in two steps. First, a variable transformation from *x* to *p* is performed by simply dividing by the population, *N*,

$$\begin{array}{c} p=\frac{x}{N} \end{array}$$ (16)

Then, recognizing that *σ*(*x*, *d*) must be strictly greater than *βx*_*i*_ *x*_*s*_ over the entire state space, we have

$$\begin{array}{c} \pi \left(x,d\right)=1-\frac{\sigma (x,d)}{\beta x_{i}x_{s}} \end{array}$$ (17)

Substituting Eqs (16) and (17) into Eq (14) results in *Q* = ∇_*x*_ *f*(*x*, *d*). With this equality, Eqs (15) and (9) are equivalent and solve for the same control, d*. When $d=\hat{d}=d^{*}$, the population disturbance is optimal and the individual will do no better by deviating. This result is useful, because it enables solving the game using only Bellman optimality.

It remains to specify the objective functions in the Bellman equation. We propose two objective functions to model activity cascade participation:

1. **Zero constant**: Assume no running cost, and cascade growth is governed by a static transmission ratio, *β*.
  $$\begin{array}{ccc} g_{\sigma }\left(x,d\right) & = & 0\\ \sigma (x,d) & = & 0 \end{array}$$
  This is the trivial case where *d* has no influence on the SIR dynamics, and the cascade is left to grow (and eventually decay) exponentially depending solely on parameters, *β* and *γ*. For completeness, the value function associated with this objective function is ∀*x* ∈ *X*, *V*(*x*) = 0. This objective function captures cascade growth with minimal interference.
2. **Expectation driven**: The cost objective is governed by a plan of an expected outcome in the cascade.
  $$\begin{array}{ccc} g_{\sigma }\left(x,d\right) & = & {∥x-\psi (x)∥}^{2}\\ \sigma (x,d) & = & (-\beta x_{s}x_{i}+d\max\{\text{sign}(x_{s}),0\})\xi \\ \xi & = & {\left[\begin{array}{ccc} 1 & -1 & 0 \end{array}\right]}^{T} \end{array}$$
  where *ξ* is used as a placement term and *ψ* is a state map. In this work, many of the cascades exhibit a macro-level linear fit such that this choice of *σ*(*x*, *d*) yields a uniformly distanced *ψ*(*x*) sequence over *x*. When solving for *d* using FMSL methods, *ψ* is implemented as a sequence of desired target points obtained empirically from data.

**Fig 3:**
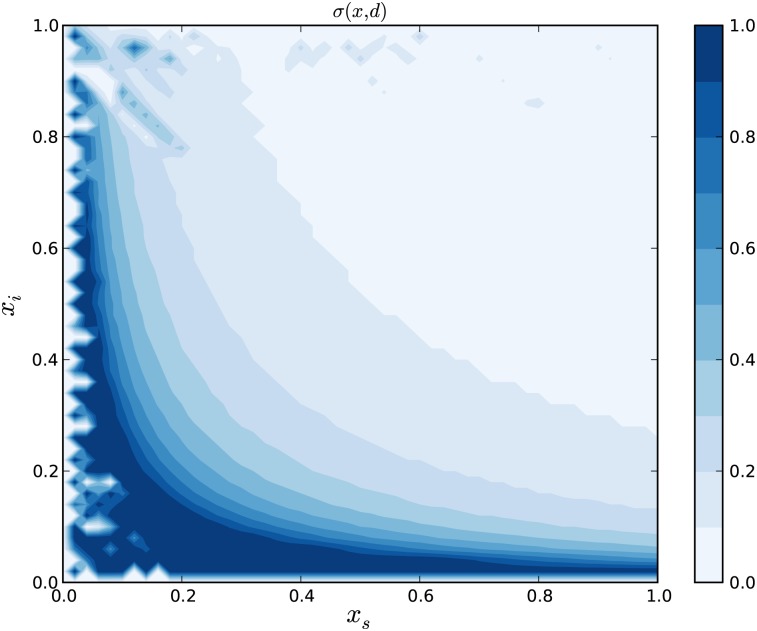
This is the optimal disturbance, *σ*(*x*, *d*), over the state space, *X*. In this diagram, the state space and output have been normalized between 0 and 1. The shape of the control corresponds to increased control effort when the value of either *x*_*s*_ or *x*_*i*_ approaches 0. If this control is used by the modeled population, then the state will eventually reach the target set, 𝒯.

The optimal disturbance function over a normalized population for candidate function (2) is obtained using the FMSL algorithm [41]. The result is shown in Fig 3, where the recovered state is removed from the phase portrait because of its zero control impact in the *ξ* matrix. This phase plot of *σ* over *x*_*s*_ and *x*_*i*_ shows a steep increase in the disturbance term as the number of susceptible and infected individuals increase. This makes intuitive sense for protest cascades, because it should require more effort to grow a cascade with fewer current participants, or when the pool of potential new participants is small.

To use this result as a practical feature for cascade growth forecasting, we create a new combined *σ* function

$$\begin{array}{c} \sigma \left(x,\eta ,\nu \right)=(-\beta \eta x_{s}x_{i}+\max\{\eta \nu \text{sign}(x_{s}),0\})\xi \end{array}$$ (18)

where *η* is a parameter describing the degree to which a population behaves using running cost candidate functions (1) or (2). In this combined form, *d* = *ην*. This we term the cost of the cascade, because it represents the amount of effort that a population must enact through *σ* in order to deviate from the SIR dynamic model, *f*_*SIR*_ and accruing cost based on the objective function in Eq (7).

### Estimating Cost Parameters from Data

Having formulated the objective function and derived the cost term, *d*, we now describe how the cost feature for cascade growth can be calculated from data. A cascade is a time series sequence of tweets,

$$\begin{array}{c} \hat{s}:\{s^{0},s^{1},\ldots ,s^{I}\} \end{array}$$ (19)

where ${\hat{s}}^{i}$ is the empirical measurement of cumulative totals Eq (4) of the number of tweets in the cascade since inception. Each point in the sequence represents the cumulative total number of tweets ${\hat{s}}^{i},i\leq I$ that have participated in the cascade at time *t* ^*i*^, *i* ≤ *I*. The length of the sequence is *I* with index *i*. The associated time sequence,

$$\begin{array}{c} t:\{t^{0},t^{1},\ldots ,t^{I}\} \end{array}$$ (20)

represents the posting times of each tweet.

Using the SIR model with cost disturbance introduced in Eq (5), the sequence approximating the cascade is given by

$$\begin{array}{c} s_{\eta ,\nu ,\beta }\left(t^{i}\right)=x_{s}\left(0\right)-\int_{0}^{t^{i}}f_{SIR}+\sigma \left(x,\eta ,\nu \right)dt \end{array}$$ (21)

where *f*_*SIR*_ has the form of Eq (3) and *f*_*D*_ = *σ*(*x*, *η*, *ν*), respectively. The initial condition, *x*_*s*_(0) is approximated from data (i.e. cumulative user degree). The term, *s*_*η*, *ν*, *β*_, signifies that this sequence approximates *s* numerically with parameters *η*, *ν*, and *β*. In practice, numerically integrating Eq (21) with Eq (18), induces a high frequency noise component in the system that is not actually present because of the signum function. A scaling term, *ϕ*_*a*, *b*_(*x*_*s*_), is incorporated

$$\begin{array}{c} \sigma \left(x,\eta ,\nu \right)=\frac{1}{2}\phi_{a,b}\left(x_{s}\right)(-\beta \eta x_{s}x_{i}+\max\{\eta \nu \text{sign}(x_{s}),0\})\xi \end{array}$$ (22)

and dampens the oscillations as *x*_*s*_ → 0 using the logistic function,

$$\begin{array}{c} \phi_{a,b}\left(x_{s}\right)=\frac{1}{1+e^{-a(x_{s}-b)}} \end{array}$$ (23)

where the parameters *a* and *b* are specific to the numerical solver used.

We want to minimize the error between the empirical cascade, $\hat{s}$ and the numerically approximated cascade, *s* for *i* ≤ *I* with the error function,

$$\begin{array}{c} e_{\hat{s}}=\sum_{i=1}^{I}[\frac{{\left({\hat{s}}^{i}-s_{\eta ,\nu ,\beta }\left(t^{i}\right)\right)}^{2}}{{\hat{s}}^{i}}] \end{array}$$ (24)

The best estimate of the cost parameter is

$$\begin{array}{c} \hat{\text{cost}}=d=\eta \nu \end{array}$$ (25)

where the parameters η* and ν* are from the minimization

$$\begin{array}{c} \nu^{*},\eta^{*},\beta^{*}=\underset{\eta ,\nu ,\beta }{arg min}e_{\hat{s}}\left(s_{\eta ,\nu ,\beta }\right) \end{array}$$ (26)

**Fig 4:**
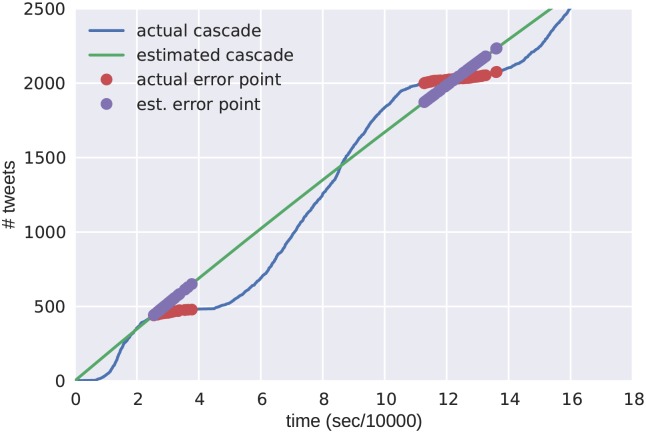
An example the optimization points chosen from an empirical sampling of a protest cascade. The green line shows the empirical cascade. The red markers indicate the measured points used in the approximation. The blue line shows the cascade approximation that minimizes Eq (26). The blue markers indicate the points used for the minimization.

To implement the minimization in Eq (26) we use the L-BFGS-B optimizing routine. Using the entire cascade is not always best for a longer term analysis, because of the daily periodic nature of the cascade volume. We use periodic sampling, and select points when the approximate derivative is 0. This is shown in Fig 4, where the markers indicate points of optimization.

## 3 Results and Discussion

We apply our methods to protests occurring in both Brazil and Venezuela in June and November of 2013. Our overall dataset from Twitter consists of over 2 million users and 40 million tweets originating from South America. Using Datasift’s streaming API, we filtered the tweets using a protest language vocabulary from [5]. The complete word list is given in the supplementary data, and contains words such as “vandalismo” and “protesta”. The geocoding component in [5] ensured that the tweets originated from the intended countries. Only tweets timestamped in June 2013 and November 2013 were included for the respective countries. To cull the activity cascades, we superimposed the tweets on the follower network obtained by querying the Twitter API. Tweets were added to the cascade only when users on the same follower network tweeted using a keyword within a duration, Δ. We set Δ = 4hrs, because this experimentally showed to be the best interval for observing information propagation for events related to the protests. This process resulted in a Brazilian protest data set containing 7291 cascades, and a Venezuelan protest data set containing 4885 cascades.

**Fig 5:**
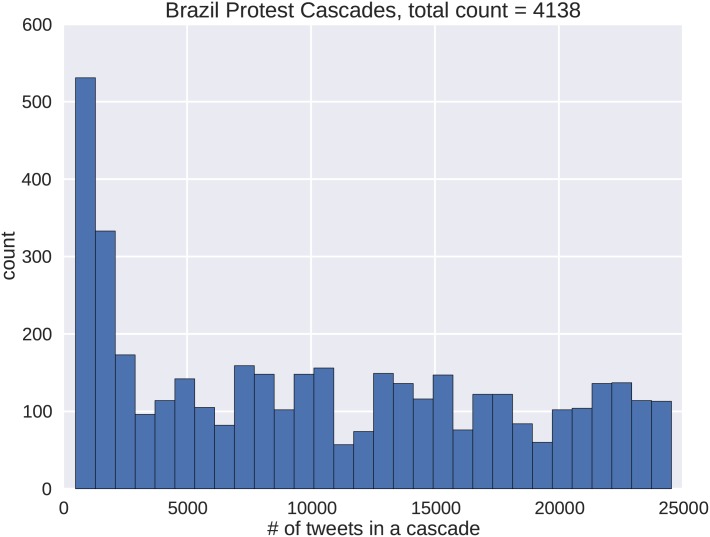
The frequency distribution shows the size ordering of Brazilian protest cascades by number of tweets used in the analysis. There are large numbers of smaller cascades followed by fewer numbers of larger cascades as indicated by the right skewing.

**Fig 6:**
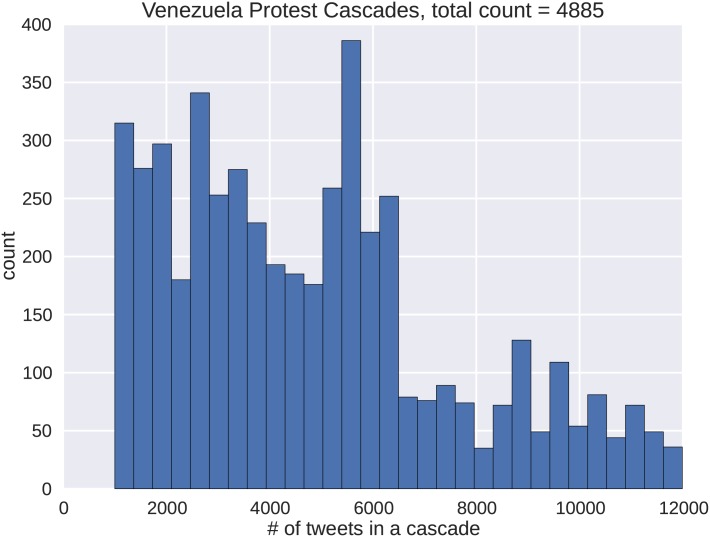
The frequency distribution shows the Venezuelan protest cascades used in this analysis ordered by the number of tweets. Compared to the Brazilian protest cascades, the relative drop in sizes between the shorter cascades and longer cascades lasts further into the median of the data set.

We only consider cascades with tweet volumes in excess of 500 tweets. The distributions for both the Brazil and Venezuela protests are shown in Figs 5 and 6 respectively. Roughly 25% of the cascades from Brazil have below 2500 tweets, or 10% of the largest tweet volume recorded. The remaining tweet volumes reach counts of around 100. In the Venezuelan dataset, roughly 75% of the cascades have below 6500 tweets or 50% of the largest tweet volume recorded. Both data sets exhibit right skewing that shows prevailing numbers of smaller cascades. However, in the Brazilian data, a given cascade is less likely to mature to the largest size. Another aspect to the skewness is that many of the cascades can be subgraphs of each other. For both protest data sets, many of the smaller cascades represent the early temporal dynamics of the larger cascades. This property of our data set is advantageous, because it enables the capture of all portions of cascade growth.

Our theoretical analysis is evaluated with the following questions:

1. **Is the more generalized cost feature parameter able to discriminate between different modes of growth, (i.e., identify** *behavior* **)?**
2. **How many initial tweets are needed to classify cascade growth and behavior?**

### Clustering Protest Cascades to Identify Behavior

**Fig 7:**
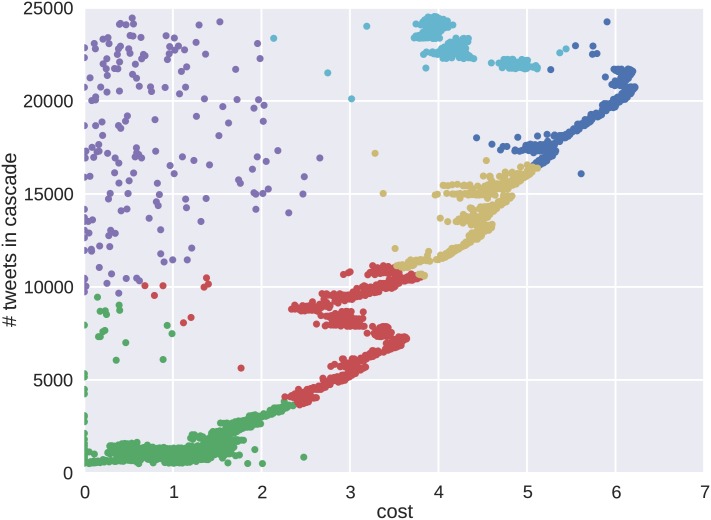
These are the clusters formed for the Brazilian protest dataset. With this dataset, the regions show a direct correlation to the number of tweets. Here, the higher cost cascades appear to result in more tweets. The apparent uncorrelated cluster of points with varying tweet counts and cost is due to random effects of the optimization algorithm used to determine the cost value for the cascades.

We answer the first question by clustering different cascade behaviors. The cost feature represents the propensity of individuals to participate in joining information chains through social networking channels. Lower cost is representative of a more epidemic style of spreading behavior, and a higher cost is indicative of more effort (outside interference) in the process. To show the relationship between cost and final cascade volume, clusters of individual cascade properties are identified on the tweet cost-volume plane. Beginning with the Brazilian protests, k-means clustering was used to form the clusters shown in Fig 7. Each color identifies one of the six clusters, where the choice of k was validated using the silhouette score and mean distance metrics.

The results show a mostly positive linear relationship between the cost and number of tweets. Clusters representing increased levels of cost indicate an increased cascade tweet volume. For the Brazilian protests, more outside intervention and deviation from epidemic spreading shows more cascade growth. The larger subgraphs of protest cascades did not favor dynamics of the epidemic model as much as the smaller subgraphs. However, given that many of the cascades are subgraphs of a larger cascade graph indicates an initial prevalence among all cascades for the lower cost behaviors. The data also show a less dense and noisy cluster located in the upper left corner that results from the increased generality of the cost feature.

**Fig 8:**
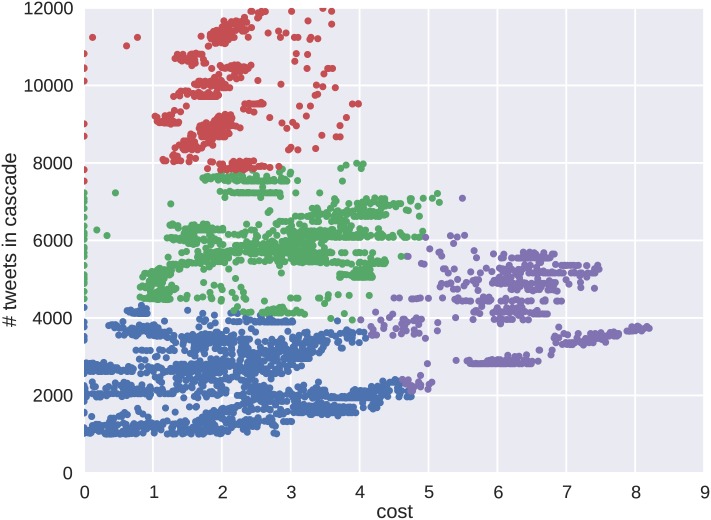
A visual inspection of the resulting clusters shows that the Venezuelan protests behave remarkably different than in Brazil. Most notably, the higher tweet count cascades appear in the lower cost ranges. Because the lower cost extends to the larger cascades in terms of tweet volume, these cascades exhibit more of the SIR dynamic behavior associated with information spreading.

Similarly for the Venezuelan protests, clusters were formed on the cost-volume plane. The results in Fig 8 show four clusters emerging from the data where *k* = 4 was validated using both the silhouette and mean distance metrics. Unlike the Brazilian protests, the clusters seen for Venezuela do not show the same level of correlation between tweet volume and cost. The Venezuelan protest cascades exhibit a much wider variety of initial behavior that converges toward a neutral range cost value as the cascades mature to the larger tweet values. This is seen in the top cluster appearing with a red color label.

**Fig 9:**
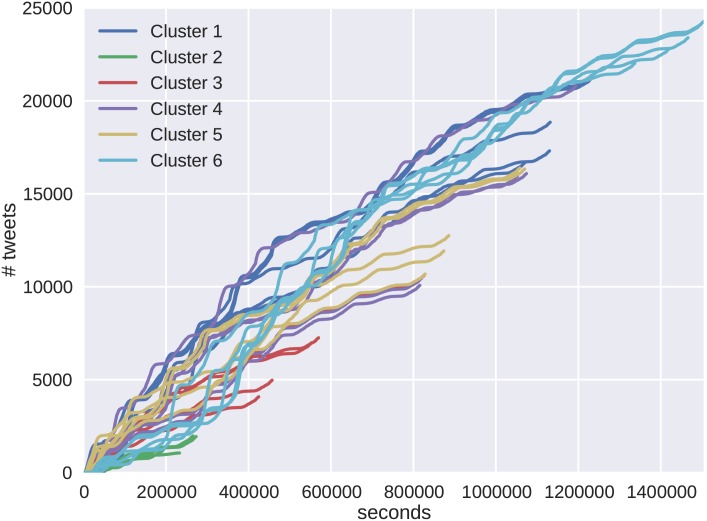
Five randomly selected cascades were chosen from each cluster to illustrate their appearance and behavior. One of the reasons we see that high linear correlation in the cluster diagram is that many of the clusters exhibit similar behavior. For protest cascades in Brazil, this means that the cascades start strong and weaken as time increases, exhibiting less SIR growth dynamics. As the cascades grow, less SIR dynamics are seen, giving them a higher cost and strong linear relationship to tweet size.

**Fig 10:**
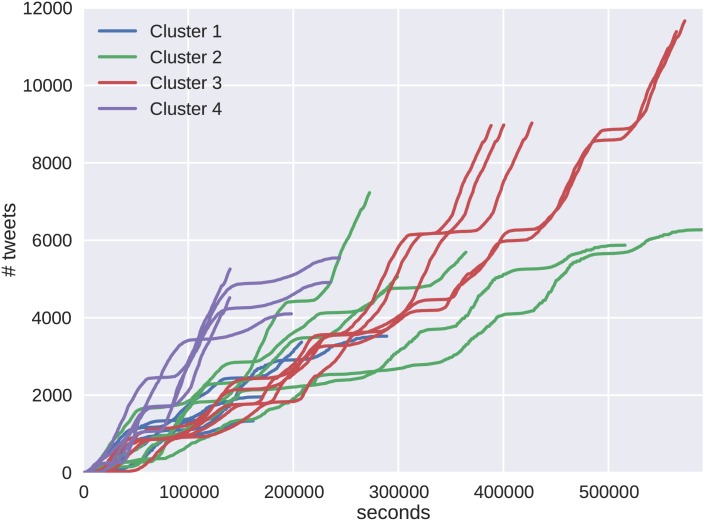
Five randomly selected cascades were chosen from each of the Venezuelan protest cascade clusters. These cascades, as opposed to the Brazilian cascades, exhibit fairly similar behavior throughout the duration of the cascade. In this protest we see more evidence of SIR dynamics, except in the cascades that make such an immediate initial rise.

The cluster results show unique differences between the cascade behaviors of the two protests. A random sampling of cascades from both protests are shown in Fig 9 for Brazil and Fig 10 for Venezuela. The color scheme of the cascades corresponds to the representative clusters in Figs 7 and 8. The behavior emerging in the clusters is verified by the shape of the resulting cascade time series. For Brazil, the clusters show varying degrees of tweet volume, but each instance shows an initial behavior with epidemic growth in tweet volume. These cascades then decrease accruing tweet volume as their growth progresses. The Venezuelan data set shows more similarity in tweet volume, but differences in behavior are seen in the rates at which the cascades accrue volume.

Clustering the data shows that there are behavioral differences that provide distinguishing characteristics for each protest dataset on the tweet cost-volume plane. The growth behavior seen in the cascades, and represented by cost, is intrinsic to many common structural and temporal features for cascade analysis. Characterizing this behavior as a cost feature and clustering similar attributes provides a measure for identifying similar trends in the activity cascade data.

### Forecasting Cascade Growth and Behavior

We build on the framework in [26, 27, 32], and now cast our forecasting question as a classification problem. Namely, if we observe an early portion of the cascade, can we forecast if it will garner significant recruits, and how will this growth behavior manifest? It is well known that information propagation on the Twitter network is significantly affected by two components: the underlying network structure around the participants of a cascade and the temporal properties of the information. Common structural and temporal features, as well as those associated with the new cost feature are shown in Table 1. We use these features in a support vector machine (SVM) classification algorithm [43] to answer the forecasting question posed above.

**Table 1:** Table showing features used for cascade forecasting. Structural and temporal features are conventional methods with which to conduct cascade forecasting. We propose the addition of the cost features listed at the bottom of the table. Here, the cost has been separated out into its constituent components, *ν* and *η*. The other parameters governing the epidemic curve, and are estimated with the fit of the cascade model are included as *β* and *γ*.

**Structural**		
S.1	Degree	# connections of the i^th^ tweet
S.2	Induced Degree	# connections of the i^th^ tweet from the first k tweets
S.3	Active Nodes	# total tweets reachable from the first k tweets
S.4	Original Connections	# neighbors of the *k* tweets who are not part of the cascade
S.5	Subgraph	# edges on the induced sub-graph of the first k participants
S.6	Border Nodes	# nodes immediately reachable from the participating nodes
S.7	User Left	# of the first k tweets not neighbors of the root
**Temporal**		
T.1	# Views	Number of users who saw the first k tweets
T.2	Avg. Reshare First	Average time between posts for the first *k*/2 tweets
T.3	Avg. Reshare Last	Average time between posts for the last *k*/2 tweets
T.4	Elapsed Time	Change in times between the orig. tweet and *i* < *k* tweets
T.5	Change dt	Change in time between tweets
**Cost**		
C.1	*β*—transmission rate	From the *SIR* epidemiological model
C.2	*γ*—recovery rate	From *SIR* epidemiological model
C.3	*ν*—participation percentage	% users participating in the forced cascade
C.4	*η*—cost estimate	Number of users attained per unit time

From our initial studies in trying to forecast only tweet volume doubling, we indeed found corroborating evidence that the structural and temporal properties of the protest cascades perform with higher precision and recall than a base rate as initially shown in [32]. Our study differs, because it includes cost attributes identifying the behavioral cluster describing cascade growth. Cost as a cascade feature is a more generalized parameter version of the temporal features which include the elapsed time of *every* tweet, *i* < *k*, ∀*k* ≤ *I*, for the first *k* tweets in a cascade. Therefore, we limit our cost feature comparison between elapsed temporal and cost features. We also note the added benefit of achieving reasonable performance with only temporal features, because expensive structural feature calculations can be avoided. For comparison, calculating the structural features consumed up to 500GB of memory and took on the order of days to accomplish. In contrast, the temporal feature calculations took place in hours on a consumer level machine with only 4GB of memory. The difference lies in the number of data points used for each calculation. The structural features require iteration over the follower graph with nodes on the order of millions and edges on the order of billions that scale with the size of the cascade. Temporal features only include a point for each timestamp in the cascade.

**Fig 11:**
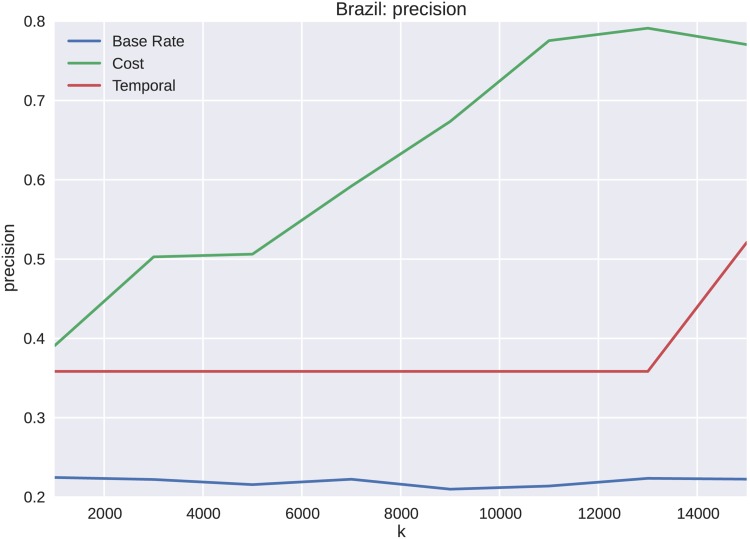
Precision for the Brazilian data set is given as a function of *k*. As expected, the more observations available for a given cascade, the better forecasting ability as seen by an increasing average across each of the clusters. Convergence is seen toward 80% precision.

**Fig 12:**
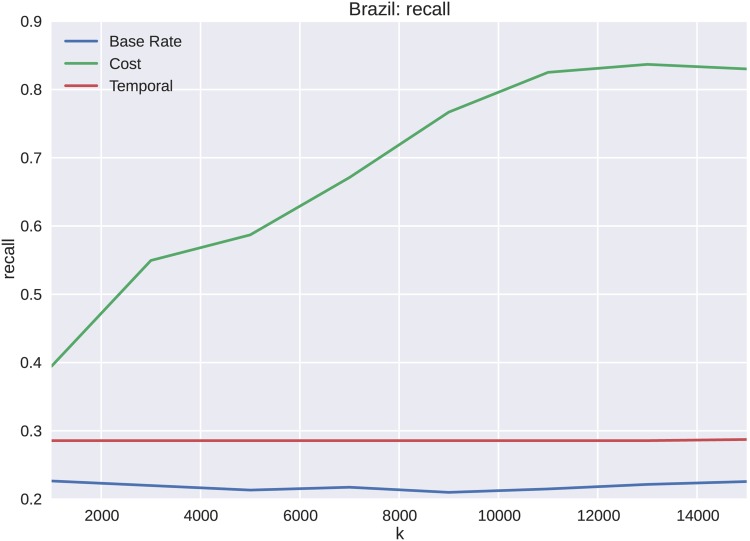
The weighted average of recall for the Brazilian data set as a function of *k* is shown. More initial observations result in better forecasting ability, and convergence is seen with increasing k toward 80%.

**Fig 13:**
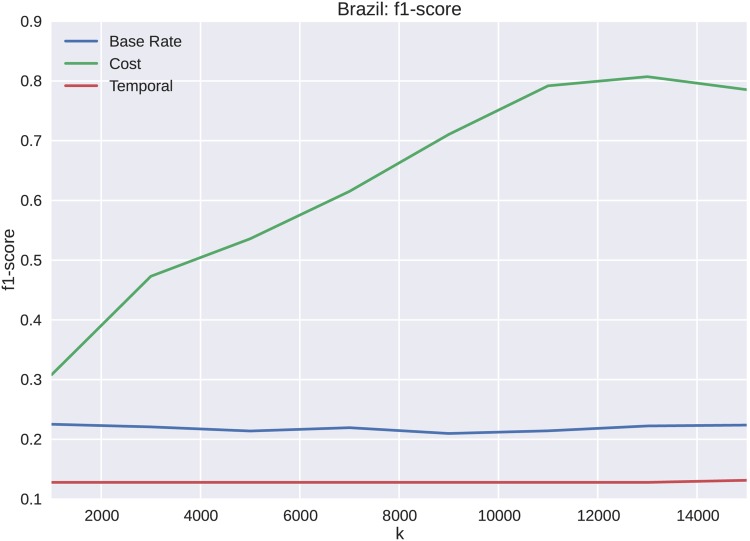
F1 score for Brazil. The cost features perform better than the base rate or temporal features, because cost includes more of they dynamic attributes of the protest cascade in the parameter set.

The dataset for the Brazilian protests is analyzed for varying lengths, *k*, of the cascades starting from the initial tweet. The precision, shown in Fig 11, shows increases from *k* = 1000 to *k* = 11000. We see similar results for both recall and the weighted F1 score shown in Figs 12 and 13 respectively. These metrics show substantial improvement over both the sample frequency base rate and temporal features. One of the primary reasons that the temporal features do not forecast membership of a cascade to a particular cluster as well is that the elapsed time temporal feature over-fits the cascades. When only a portion of the cascades are seen, the clusters become indistinguishable. The cost captures more of the cascade dynamics, and discriminates at an earlier stage of cascade growth. This effect only becomes more exaggerated as k is increased to include more cascade volume in the forecast.

**Fig 14:**
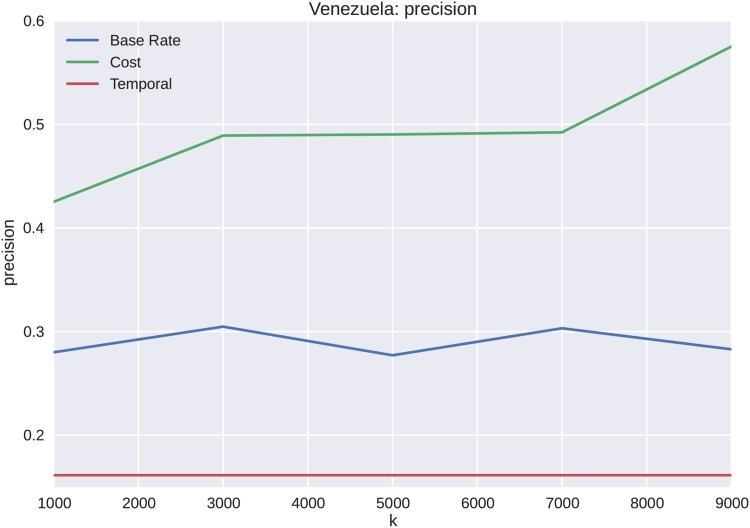
The precision scores for the Venezuelan protests are similar to that of the Brazilian protests, with an increasing precision with cost features. The base rate and temporal features do not exhibit an increase with an increase in k with respect to cluster identification ability.

**Fig 15:**
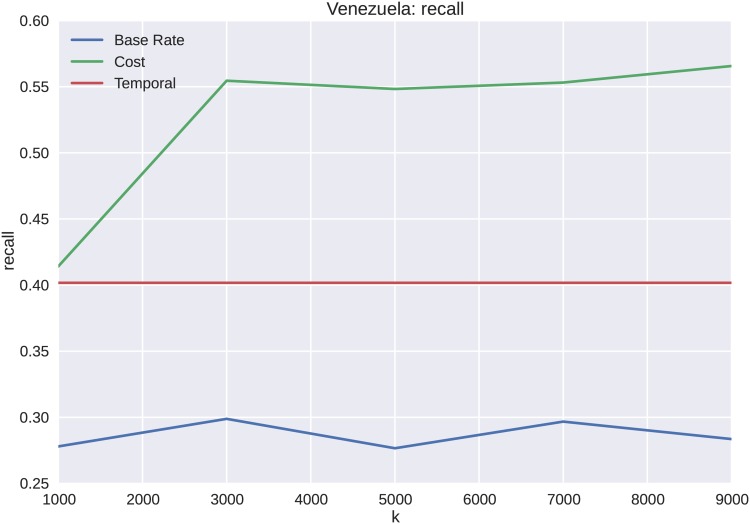
Recall scores also behave similarly to the Brazilian protests, with the exception of a higher recall than would be expected. This is an artifact of the cluster topology where the temporal features tend to forecast cascades in the lower volume cluster, which is where most of the cascades reside.

**Fig 16:**
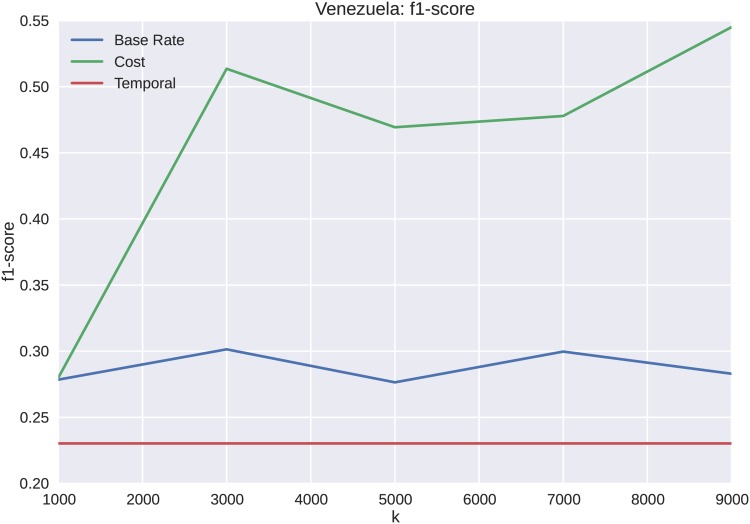
The F1 score for Venezuela forecasting shows similar results to Brazil with respect to an increasing score with increased k, when the other features exhibit no increase. The clustering approach increases in forecasting ability as the number of points increases, because it is able to discern more of the trajectory family that belongs to particular clusters.

The forecasting results for Venezuela appear similar to those of the Brazilian protest data, except with overall less precision and recall as shown in Figs 14 and 15, respectively. The reason for less overall accuracy in forecasting ability, even with a higher *k*, is that the distinction between cascade volume and cost is not as well defined for Venezuela as it is for Brazil. However, as shown in Fig 16, the overall performance of cost features to temporal features is superior for all values of *k*.

These results are significant for two reasons. First, the cost feature is able to identify membership of a cascade to clusters of different growth behavior better than the base rate or temporal features. It is able to do this for both the Brazilian and Venezuelan datasets which provide supporting evidence for its ability to discriminate among different cascade growths. Second, the cost feature does not require the extensive computational resources like the structural features. The advantage here is that the cost feature information size does not need to scale with *k*, because a cascade of any length is described by the same number of cost parameters.

## 4 Conclusions

A cost feature capturing protest cascade growth in Twitter was developed using a differential game model. This cost represents the deviation an individual may take from what would be the exponential cascade growth modeled as an SIR epidemic curve. The differential game is solved by showing that an individual can do no better than the population dynamics, and then fitting this approximate model to empirical data from both Brazilian and Venezuelan protest cascades. We found that using cost as a forecasting feature shows distinct cluster profiles for both protest events. Furthermore, we are able to forecast cascade growth dynamics and volume by identifying the cluster a cascade most likely belongs to using a support vector machine. Despite these results, cascade growth is still subject to sensitivity in the antecedents and a wide range of fluctuations in the consequents making it subject to noise and time dependencies.

Toward future research, there are a number of ways to characterize cascade growth. This cost model approach is just one way to parameterize cascade growth behavior for better interpretation. Using it in the context of transfer learning with tools such as Dirichlet processes (i.e. [44]) could potentially improve the performance of these methods for producing an even more general approach to forecasting Twitter cascade behavior for protests. Despite any other number of possible improvements or extensions, the crux of the problem remains that of finding the invariant set of features from which to understand cascade growth across multiple domains.

## References

[1] Mao H, Counts S, Bollen J (2011) Predicting financial markets: Comparing survey, news, twitter and search engine data. arXiv preprint arXiv:11121051.

[2] Ruiz EJ, Hristidis V, Castillo C, Gionis A, Jaimes A (2012) Correlating financial time series with micro-blogging activity. In: Proceedings of the Fifth ACM International Conference on Web Search and Data Mining. New York, NY, USA: ACM, WSDM’12, pp. 513–522. URL http://doi.acm.org/10.1145/2124295.2124358

[3] Lee K, Agrawal A, Choudhary A (2013) Real-time disease surveillance using twitter data: demonstration on flu and cancer. In: Proceedings of the KDD’13. pp. 1474–1477.

[4] Achrekar H, Gandhe A, Lazarus R, Yu SH, Liu B (2011) Predicting flu trends using twitter data. In: Computer Communications Workshops (INFOCOM WKSHPS), 2011 IEEE Conference on. pp. 702–707.

[5] Ramakrishnan N, Butler P, Muthiah S, Self N, Khandpur R, et al. (2014)’beating the news’ with embers: Forecasting civil unrest using open source indicators. In: Proceedings of the 20th ACM SIGKDD International Conference on Knowledge Discovery and Data Mining. New York, NY, USA: ACM, KDD’14, pp. 1799–1808. URL http://doi.acm.org/10.1145/2623330.2623373

[6] Hua T, Lu CT, Ramakrishnan N, Chen F, Arredondo J, et al (2013) Analyzing civil unrest through social media. IEEE Computer 46: 80–84. 10.1109/MC.2013.442

[7] Braha D (2012) Global civil unrest: Contagion, self-organization, and prediction. PLoS ONE 7: e48596 10.1371/journal.pone.0048596 23119067 PMC3485346

[8] Oliver PE, Myers DJ (1998) Diffusion models of cycles of protest as a theory of social movements. Presented at the Congress of the International Sociological Association.

[9] Gonzlez-Bailn S, Borge-Holthoefer J, Rivero A, Moreno Y (2011) The dynamics of protest recruitment through an online network. Scientific Reports 1. 10.1038/srep00197 PMC3240992 22355712

[10] Saad-Filho A (2013) Mass protests under left neoliberalism: Brazil, june-july 2013. Critical Sociology 39: 657–669. 10.1177/0896920513501906

[11] Morales A, Borondo J, Losada J, Benito R (2014) Efficiency of human activity on information spreading on twitter. Social Networks 39: 1–11. 10.1016/j.socnet.2014.03.007

[12] Bond RM, Fariss CJ, Jones JJ, Kramer ADI, Marlow C, et al (2012) A 61-million-person experiment in social influence and political mobilization. Nature 489: 295–298. 10.1038/nature11421 22972300 PMC3834737

[13] Conover MD, Ferrara E, Menczer F, Flammini A (2013) The digital evolution of occupy wall street. PLoS ONE 8: e64679 10.1371/journal.pone.0064679 23734215 PMC3667169

[14] Galuba W, Aberer K, Chakraborty D, Despotovic Z, Kellerer W (2010) Outtweeting the twitterers—predicting information cascades in microblogs. In: Proceedings of the 3rd Wonference on Online Social Networks. Berkeley, CA, USA: USENIX Association, WOSN’10, pp. 3–3. URL http://dl.acm.org/citation.cfm?id=1863190.1863193

[15] Winters M, Weitz-Shapiro R (2014) Partisan protesters and nonpartisan protests in brazil. Journal of Politics in Latin America 6: 137–150.

[16] Hidalgo M (2014) The 2012 and 2013 presidential elections in venezuela. Electoral Studies 34: 315–321. 10.1016/j.electstud.2013.12.007

[17] Kermack WO, McKendrick AG (1927) A contribution to the mathematical theory of epidemics. Proceedings of the Royal Society of London A: Mathematical, Physical and Engineering Sciences 115: 700–721. 10.1098/rspa.1927.0118

[18] Goel S, Watts DJ, Goldstein DG (2012) The structure of online diffusion networks. In: Proceedings of the 13th ACM Conference on Electronic Commerce. New York, NY, USA: ACM, EC’12, pp. 623–638. URL http://doi.acm.org/10.1145/2229012.2229058

[19] Kimura M, Saito K, Nakano R, Motoda H (2010) Extracting influential nodes on a social network for information diffusion. Data Mining and Knowledge Discovery 20: 70–97. 10.1007/s10618-009-0150-5

[20] Guille A, Hacid H (2012) A predictive model for the temporal dynamics of information diffusion in online social networks. In: Proceedings of the 21st International Conference on World Wide Web. New York, NY, USA: ACM, WWW’12 Companion, pp. 1145–1152. URL http://doi.acm.org/10.1145/2187980.2188254

[21] Hackett A, Melnik S, Gleeson JP (2011) Cascades on a class of clustered random networks. Phys Rev E 83: 056107 10.1103/PhysRevE.83.056107 21728605

[22] Nematzadeh A, Ferrara E, Flammini A, Ahn YY (2014) Optimal network modularity for information diffusion. Phys Rev Lett 113: 088701 10.1103/PhysRevLett.113.088701 25192129

[23] West BJ, Grigolini P (2011) Complex Webs. Cambridge: Cambridge University Press.

[24] Bakshy E, Karrer B, Adamic LA (2009) Social influence and the diffusion of user-created content. In: Proceedings of the 10th ACM Conference on Electronic Commerce. New York, NY, USA: ACM, EC’09, pp. 325–334. URL http://doi.acm.org/10.1145/1566374.1566421

[25] Tsur O, Rappoport A (2012) What’s in a hashtag?: Content based prediction of the spread of ideas in microblogging communities. In: Proceedings of the Fifth ACM International Conference on Web Search and Data Mining. New York, NY, USA: ACM, WSDM’12, pp. 643–652. URL http://doi.acm.org/10.1145/2124295.2124320

[26] Hong L, Dan O, Davison BD (2011) Predicting popular messages in twitter. In: Proceedings of the 20th International Conference Companion on World Wide Web. New York, NY, USA: ACM, WWW’11, pp. 57–58. URL http://doi.acm.org/10.1145/1963192.1963222

[27] Jenders M, Kasneci G, Naumann F (2013) Analyzing and predicting viral tweets. In: Proceedings of the 22nd International Conference on World Wide Web Companion. Republic and Canton of Geneva, Switzerland: International World Wide Web Conferences Steering Committee, WWW’13 Companion, pp. 657–664. URL http://dl.acm.org/citation.cfm?id=2487788.2488017

[28] Szabo G, Huberman BA (2010) Predicting the popularity of online content. Commun ACM 53: 80–88. 10.1145/1787234.1787254

[29] Osborne M, Lavrenko V (2011) RT to Win! Predicting Message Propagation in Twitter. Artificial Intelligence: 586–589.

[30] Backstrom L, Huttenlocher D, Kleinberg J, Lan X (2006) Group formation in large social networks: Membership, growth, and evolution. In: Proceedings of the 12th ACM SIGKDD International Conference on Knowledge Discovery and Data Mining. New York, NY, USA: ACM, KDD’06, pp. 44–54. URL http://doi.acm.org/10.1145/1150402.1150412

[31] Romero DM, Tan C, Ugander J (2013) On the interplay between social and topical structure. In: Proceedings of the Seventh International Conference on Weblogs and Social Media.

[32] Cheng J, Adamic LA, Dow PA, Kleinberg JM, Leskovec J (2014) Can cascades be predicted? In: Proceedings of he International Conference of WWW. URL http://arxiv.org/abs/1403.4608

[33] Easley D, Kleinberg J (2010) Networks, Crowds, and Markets: Reasoning about a Highly Connected World. Cambridge University Press.

[34] von Neumann J, Morgenstern O (1944) Theory of games and economic behavior. Princeton University Press.

[35] Isaacs R (1965) Differential Games. New York, NY: John Wiley and Sons, Inc.

[36] Merz A (1972) The game of two identical cars. Journal of Optimization Theory and Applications 9: 324–343. 10.1007/BF00932932

[37] Başar T, Olsder GJ (1999) Dynamic Noncooperative Game Theory. Number 23 in Classics in Applied Mathematics. SIAM, 2 edition.

[38] Reluga TC (2010) Game theory of social distancing in response to an epidemic. PLoS Comput Biol 6: e1000793 10.1371/journal.pcbi.1000793 20523740 PMC2877723

[39] Koku PS, Akhigbe A, Springer TM (1997) The financial impact of boycotts and threats of boycott. Journal of Business Research 40: 15–20. 10.1016/S0148-2963(96)00279-2

[40] Bertsekas DP (2001) Dynamic Programming and Optimal Control, volume 2 Athena Scientific, 2 edition.

[41] Cristiani E, Falcone M (2006) A fast marching method for pursuit-evasion games. Communications to SIMAI Congress 1.

[42] Reluga TC (2009) An sis epidemiology game with two subpopulations. Journal of Biological Dynamics 3: 515–531. 10.1080/17513750802638399 22880898

[43] Pedregosa F, Varoquaux G, Gramfort A, Michel V, Thirion B, et al (2011) Scikit-learn: Machine learning in Python. Journal of Machine Learning Research 12: 2825–2830.

[44] Nsoesie E, Leman S, Marathe M (2014) A dirichlet process model for classifying and forecasting epidemic curves. BMC Infectious Diseases 14: 12 10.1186/1471-2334-14-12 24405642 PMC3901791

